# Factors Contributing to Obesity Among Indigenous Peoples of Canada: A Narrative Review

**DOI:** 10.1177/20543581261472119

**Published:** 2026-09-30

**Authors:** Danial Aminaei, Minhal Ahmed, Asal Ozlati, Kristin Kyomi Clemens, Arsh Jain, Sonja Reichert, Stewart Harris, Jaclyn Ernst, Louise Moist

**Affiliations:** 1 Schulich School of Medicine and Dentistry, 6221 Western University, London, ON, Canada; 2 College of Dentistry, 7235 University of Saskatchewan, Saskatoon, SK, Canada; 3 Division of Endocrinology, 25472 St. Joseph’s Health Care London, London, Ontario, Canada; 4 ICES Western, Department of Epidemiology and Biostatistics, Western University, London, Ontario, Canada; 5 Lawson Research Institute, 25472 St. Joseph’s Health Care London, London, Ontario, Canada; 6 Centre for Diabetes, Endocrinology and Metabolism, 25472 St. Joseph’s Health Care London, London, Ontario, Canada; 7 Division of Nephrology, Department of Medicine, 6221 Western University, London, ON, Canada

**Keywords:** obesity, indigenous peoples, Canada, chronic kidney disease, health equity, risk factors

## Abstract

**Purpose of Review:**

Obesity is a major and growing public health concern in Canada and an important contributor to chronic kidney disease (CKD). Indigenous Peoples in Canada experience a disproportionately high burden of obesity and CKD, driven by complex social, cultural, environmental, and biological factors. This review synthesizes the literature on factors contributing to obesity among Indigenous Peoples in Canada and discusses implications for kidney health.

**Methods:**

A narrative review was conducted using predefined search terms related to obesity, Indigenous Peoples, Canada, and CKD. Following screening in Covidence, 44 studies were selected from 131 eligible articles based on relevance and contribution to emerging themes.

**Key Findings:**

Obesity among Indigenous Peoples in Canada is influenced by an interconnected network of determinants, including dietary transition, food insecurity, socioeconomic inequities, reduced physical activity, environmental change, cultural disruption, historical trauma, systemic racism, and limited access to culturally safe healthcare. Biological and genetic factors may also contribute. These determinants increase CKD risk directly through obesity-related kidney injury and indirectly through diabetes and hypertension. Barriers to obesity treatment, including limited access to bariatric surgery, pharmacotherapy, and culturally appropriate care, may further contribute to disparities in kidney health and transplantation access.

**Limitations:**

The literature is limited by reliance on cross-sectional and self-reported data, a lack of longitudinal studies, underrepresentation of urban Indigenous populations, and limited evaluation of culturally relevant measures of health and obesity.

**Implications:**

Addressing obesity among Indigenous Peoples requires culturally grounded, community-led, and system-level interventions that improve food security, healthcare access, and culturally safe obesity management. Such approaches may contribute to reducing CKD burden and advancing kidney health equity.

## Introduction

Obesity is a global epidemic, affecting over 650 million people worldwide.^1^ In Canada, the prevalence of obesity, defined by a Body Mass Index (BMI) ≥ 30 kg/m^2^, increased by 300% between 1985 and 2016,^2^ and has significantly contributed to healthcare costs.^3^ Over the past three decades, rates of obesity have tripled, with severe obesity experiencing a four-time increase, affecting nearly 2 million Canadians.^4^

Obesity is closely associated with increased mortality (6–14-year life expectancy reduction) and the development of multiple comorbid conditions, including diabetes, cardiovascular disease, kidney disorders, depression, and anxiety.^4^ It is also accountable for nearly 20% of all cancers.^5^

Obesity impacts kidney health through multiple mechanisms. Increased body mass places a greater workload on the kidneys, increasing the glomerular filtration rate (GFR) and causing glomerular hypertension, which can damage the filtration barriers. This hyperfiltration leads to glomerulomegaly and may progress to focal segmental glomerulosclerosis, known as obesity-related glomerulopathy (ORG).^6^ Metabolically, obesity promotes insulin resistance, hyperinsulinemia, and dyslipidemia, which harm glomerular and tubular structures through lipotoxicity and excess deposition of free fatty acids and triglycerides.^6^ Excess adipose tissue also secretes pro-inflammatory and profibrotic mediators, including adipokines and cytokines such as leptin, adiponectin, TNF-α, MCP-1, and TGF-β, driving chronic inflammation, oxidative stress, endothelial dysfunction, and kidney fibrosis.^7^ Additionally, obesity activates the renin-angiotensin-aldosterone system (RAAS), causing hypertension and further kidney microvascular injury.^6^ Indirectly, obesity increases the risk of diabetes and hypertension, the two leading independent risk factors for chronic kidney disease (CKD; across G categories), thereby accelerating its progression to more advanced CKD categories (G4-G5).^6,7^

Moreover, obesity has profound implications for patient care, particularly for patients with CKD, where it affects peri-operative and post-operative morbidity and mortality, including kidney transplant outcomes.^8^ It may also delay and restrict management options. For example, over 30% of transplant candidates may be excluded from life-saving transplants due to obesity.^9^

Indigenous peoples collectively refers to First Nations, Métis, and Inuit communities as recognized in the Canadian Constitution.^10^ Emerging studies have shown that obesity disproportionately affects this population in Canada. For example, the prevalence of obesity, based on self-reported height and weight, ranges from 30% to 50% among First Nations peoples living on reserves, 21% to 42% among First Nations people living off reserves, compared to 12% to 31% in non-Indigenous populations.^11^ This has contributed to higher rates of related health complications and worsening kidney disease outcomes in these communities. In fact, previous studies have shown that Indigenous Canadians have a twofold higher prevalence of CKD, particularly in advanced categories (G3-5) compared to non-Indigenous Canadians with 25% of Indigenous Canadians diagnosed with CKD. Geographically, 34% of rural Indigenous Canadians had CKD as opposed to 18% of non-remote Indigenous Canadians.^12,13^ There has also been an association between albuminuria and increasing body mass index.^12,14^ Indigenous patients with CKD also have lower rates of kidney transplantation compared to non-Indigenous patients (27% vs. 42% receive transplants, respectively).^15^ Thus, effectively managing obesity in Indigenous populations may reduce the incidence of CKD and increase eligibility for kidney transplantation among those affected.

Obesity among Indigenous peoples in Canada is a complex and multifaceted issue. Given the significant impact of obesity on health, CKD and healthcare, understanding the underlying risk factors contributing to their high prevalence in these communities is crucial. Addressing this complexity aligns with the Truth and Reconciliation Commission’s Calls to Action, which emphasize the need to close the gaps in health outcomes between Indigenous and non-Indigenous communities.^16^

The objective of this study is to review the existing literature on the key risk factors associated with obesity and CKD in these population among Canadian Indigenous peoples. By exploring these factors, this review seeks to provide a comprehensive understanding of the issue. This understanding is essential for informing the development of effective weight management programs, policies, and patient education aimed at reducing the incidence of CKD in Indigenous communities and ensuring equitable access to kidney transplantation for those affected.

## Methods

A literature search was conducted across four databases: PubMed, Scopus, the Bibliography of Indigenous Peoples in North America, and i-Portal. The search utilized specific keywords and phrases as follows:

(

(Obesity AND (Indigenous OR “First Nations” OR Métis OR Inuit) AND Canada).

OR.

((Causes OR “Risk Factors” OR Qualitative OR “Health Disparities” OR “Cultural Factors” OR “Social Determinants”)

AND Obesity AND (Indigenous OR “First Nations” OR Métis OR Inuit) AND Canada).

)

OR.

(

(“Chronic Kidney Disease” AND (Indigenous OR “First Nations” OR Métis OR Inuit) AND Canada).

OR.

(“Chronic Kidney Disease” AND Obesity AND (Indigenous OR “First Nations” OR Métis OR Inuit) AND Canada).

)

Studies were uploaded to Covidence for systematic screening. Reviewers independently conducted title and abstract screening, followed by full-text screening. Studies were deemed eligible if they examined causes or contributing factors related to obesity and kidney health among Indigenous peoples in Canada and were published in English. Conference abstracts, editorials, commentaries, and protocols were excluded.

Of 214 eligible studies, 58 were selected for inclusion based on their relevance to the topic, unique insights, and contributions to emerging themes. This approach provided a diverse range of perspectives, reduced redundancy, and emphasized studies offering meaningful insights into the synthesis (Figure 1).

**Figure 1.**
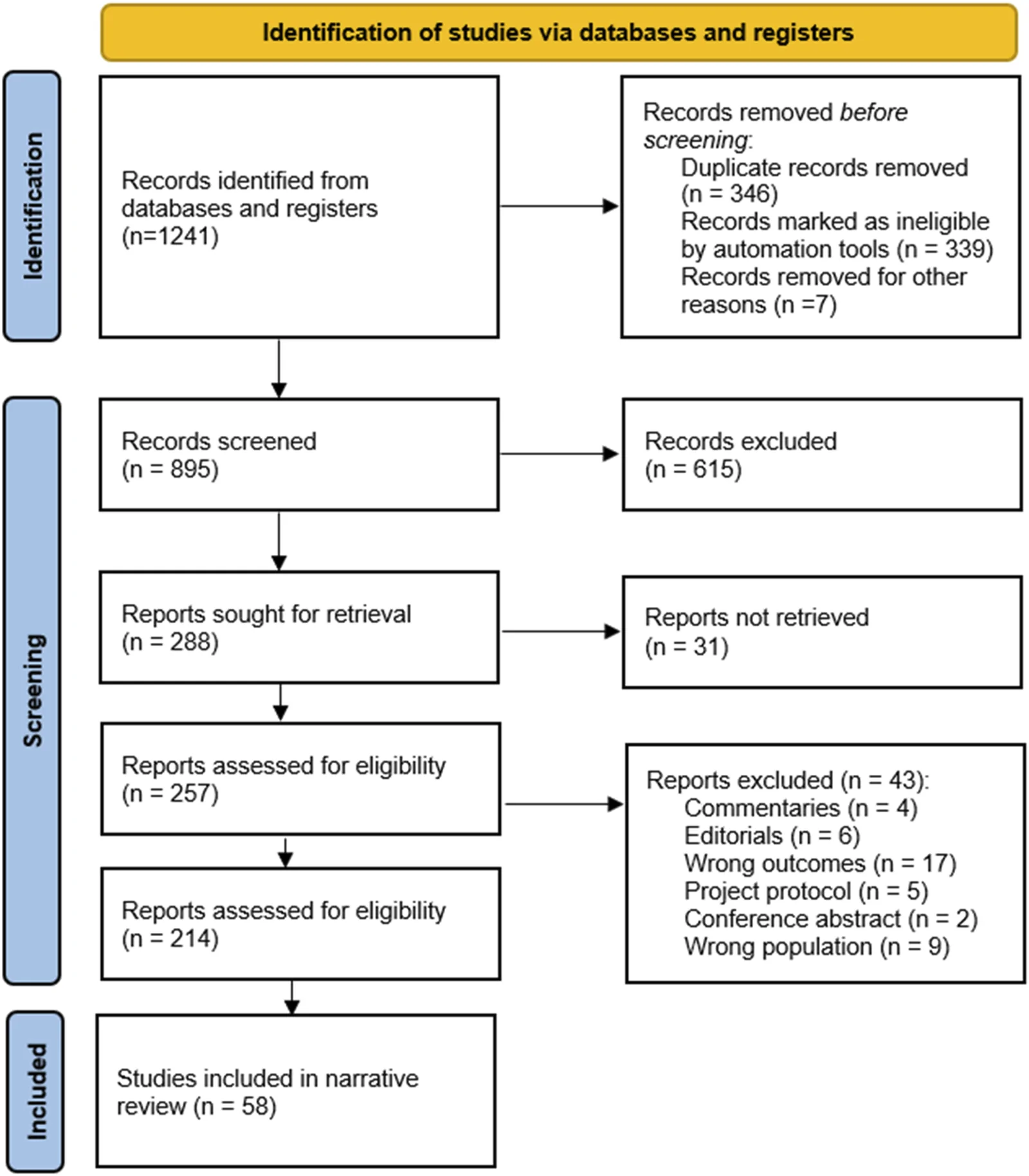
Flowchart of study selection process for the narrative review^17^

## Review

Obesity among Indigenous Peoples of Canada is driven by a multifactorial interplay of dietary transitions, physical inactivity, food insecurity, socioeconomic challenges, environmental shifts, cultural changes, and biological predispositions. These factors are deeply intertwined, resulting in disproportionately high obesity rates in Indigenous peoples. Supporting the health care of Indigenous peoples with obesity requires an understanding of the complex historical, environmental, and social contexts that influence health outcomes.

### Dietary Transition

The shift from nutrient-rich traditional diets to energy-dense market foods has played a central role in the rise of obesity among Indigenous peoples. Historically, traditional foods such as wild game, fish, and berries provided essential nutrients and supported healthy body weights.^18,19^ However, contemporary diets in many Indigenous communities are now dominated by processed foods high in fats, sugars, and refined carbohydrates. This shift is largely due to environmental changes, economic barriers, and reduced access to traditional food sources.^20,21^ In many communities, particularly in remote and northern areas, reliance on cheaper, less nutritious foods has increased.^20^ Younger generations also have less experience in food gathering activities and rely more heavily on motorized equipment. The high cost of equipment along with rules and regulations placed by the government regarding number of animals that can be hunted, limited the availability and accessibility of traditional foods such as caribou and seal.^20^

### Socioeconomic Factors

Food insecurity is a persistent challenge within Indigenous communities particularly in remote and northern areas.^22,23^ Approximately 46% of First Nation households experience food insecurity.^23^ A Canadian Community Health Survey (CCHS) in 2012 revealed a disparity in food insecurity among Indigenous households (20%) and Non-Indigenous households (8%). Similarly, First Nations Regional Health Survey Phase III revealed that 50.8% of on reserve First Nations households are food-insecure.^23^

Socioeconomic disparities, including poverty, unemployment, and reliance on social assistance, are significant contributors to food insecurity in Indigenous communities.^22,24^ Low-income households often struggle to afford healthy food options, forcing them to depend on high-calorie, processed alternatives that contribute to obesity.^25^ Geographic isolation & lack of year-round access are among other contributors.^26^ For Indigenous populations living off-reserve, proximity to “fast food” restaurants and convenience stores in low-income neighborhoods may promote unhealthy eating habits.^27^ A study found that socioeconomic disparities are important determinants of patient outcomes related to CKD in Indigenous populations.^28^ These socioeconomic disparities are especially detrimental to children, as food insecurity is strongly linked to higher risk of being overweight or obese.^25^

Beyond the risk of developing CKD, Indigenous Canadians diagnosed with CKD have poorer outcomes and require transplant more often, but do not get as many transplants as non-Indigenous Canadians. Barriers to kidney transplants in Indigenous people on kidney replacement therapy (KRT; CKD G5D and CKD G1T-G5T), including functional transplant and dialysis, include low health literacy, poor access to health care, lack of cultural safety in mainstream health services, and systemic racism and cultural misunderstanding.^29^

### Physical Inactivity and Sedentary Lifestyles

The decline in traditional physical activities, such as hunting, fishing, berry picking, and canoeing may have also contributed to increasing obesity rates in Indigenous peoples.^30^ In the past, Indigenous communities engaged in physically demanding activities essential for daily living, which naturally promoted active lifestyles.^31^ However, modern technological advancements and urbanization have resulted in more sedentary lifestyles.^20,32^ Sedentary behaviors particularly excessive screen time among youth, have been strongly linked to increased rates of obesity and chronic diseases.^33-35^ Obesity is a major driver of CKD due to glomerulomegaly, and focal and segmental glomerulosclerosis, all of which are worsened by sedentarism.^36^

### Cultural Influences

Cultural changes, driven by colonialism and acculturation, have significantly contributed to the obesity epidemic among Indigenous peoples. The erosion of traditional food systems and practices has disconnected many communities from the land and their cultural dietary practices that once supported healthy eating and active living.^37,38^ As Westernized lifestyles are increasingly adopted, traditional foods and knowledge are lost, leading to greater dependence on processed, nutrient-poor foods.^19,21^ This cultural disruption has been associated with increasing obesity and risk to type 2 diabetes mellitus, both causing CKD.^39^ This is further influenced by epigenetic factors due to the long term effect of colonialism which increases the risk of obesity and CKD as well.^40^

Cultural perceptions of body image also play a role in obesity-related behaviors. In some Indigenous communities, larger body sizes are traditionally associated with strength and health, influencing attitudes toward weight management and obesity prevention.^16^ These norms can conflict with Western ideals of thinness, leading to body image dissatisfaction and unhealthy weight control behaviors.^37^

### Environmental Influences

Environmental changes such as climate change, deforestation, and wildlife depletion further compound the challenges Indigenous communities face. The loss of traditional food sources, combined with increased reliance on market foods, negatively impacts health outcomes, driving obesity rates upward.^19,41^ Environmental contaminants like mercury and persistent organic pollutants (POPs) present in traditional foods also discourage their consumption, forcing communities to rely on less nutritious market alternatives.^41^ Exposure to POPs and mercury toxicity increased obesity and obesity-associated metabolic disturbances such as type 2 diabetes mellitus and metabolic syndrome in most populations, leading to CKD. Furthermore, many POPs and heavy metals, namely arsenic, cadmium, lead, and mercury, are potent known toxins to the kidneys and are known to increase risk of type 2 diabetes mellitus and complications. These POPs have been found in the drinking water and supplies of various Canadian Indigenous populations.^42^ It remains unknow if these POPs influence the risk of CKD progressions.

In some of the northern and remote areas, the harsh climate and long winters may limit access to fresh foods during certain seasons. For instance, traditional food practices like hunting and gathering become more difficult in winter due to deforestation and wildlife scarcity.^19,35,43^ Similarly, the harsh climate also further perpetuates physical inactivity in the lack of recreational facilities for exercise.^20,35^

### Biological and Genetic Factors

While environmental and lifestyle factors play a significant role in obesity among Indigenous peoples, biological and genetic predispositions are also among the contributors. The “thrifty genotype” hypothesis suggests that Indigenous peoples may have a genetic predisposition to obesity due to ancestral adaptations to feast-famine cycles, which make them more susceptible to weight gain in modern environments.^44^ Genetic variants such as the GNB3 825T/T genotype and the LMNA 1908T/C genotype have been linked to higher BMI, adipose deposition, and metabolic disorders in some Indigenous populations.^32,45^ Further, the presence of obesity and complications like diabetes in the population creates a vicious cycle where a mother’s intrauterine environment is hyperglycemic leading a predisposition for the child to be obese and diabetic, both of which harm the kidneys.

### BMI Measure of Body Composition

Some studies also suggest that BMI cutoffs used to classify obesity may not be fully applicable for Indigenous population. For example, individuals from an Inuit background often have fewer metabolic complications compared to non-Indigenous groups, even at higher BMI levels. Another study showed Inuit people have lower triglyceride levels and higher HDL cholesterol levels, despite having elevated BMI.^46,47^ This suggests that conventional BMI thresholds may overestimate obesity-related health risks in these populations. Adjusting BMI cut-offs to better reflect the unique metabolic profiles of Indigenous peoples could improve obesity assessments and support more targeted healthcare interventions This knowledge of body composition may also influence estimates of kidney function, another area that has been understudied in this high-risk population.

### Psychosocial Stressors and Substance Abuse

Psychosocial stressors, particularly those stemming from intergenerational trauma caused by colonization and residential schools, might contribute to obesity in Indigenous peoples. Historical trauma has increased the risk of unhealthy coping mechanisms, such as overeating and physical inactivity, which exacerbate weight gain and obesity.^38^ Indigenous children and youth who endure negative school environments, such as bullying, racism, and exposure to drugs are at a heightened risk for obesity.^25^ These psychological and social challenges often result in emotional distress that manifests through maladaptive behaviors, including substance abuse and emotional eating. Smoking is linked to unhealthy weight gain and metabolic disturbances, such as increased visceral adiposity.^48^ Alcohol consumption similarly contributes to obesity by increasing caloric intake, reducing physical activity, and impairing nutrient metabolism.^49,50^ In a similar vein, smoking raises the risk of micro- and macroalbuminuria worsening glomerular filtration rate and CKD.^33^ These high risk behaviors likely carry over to influence on nutritional intake and an active lifestyle affect cardio-kidney metabolic health.

### Bariatric Surgeries & Pharmaceutical Managements

Bariatric surgery is an important treatment option for obesity, with Indigenous patients achieving weight loss outcomes comparable to those of non-Indigenous patients.^51^ However, factors such as the need for multiple clinic visits, limited access to interpretation services, cultural communication differences, low health literacy, unfamiliar medical terminology, lack of trust in healthcare providers, and stigma surrounding obesity can lead to lower attendance and higher attrition rates.^51,52^ Indigenous patients may also experience slightly higher rates of postoperative complications, including surgical site infections and re-operations within 30 days.^53^ Engagement with bariatric programs is further hindered by the lack of culturally appropriate support, such as the absence of Indigenous healthcare workers and limited opportunities for emotional or cultural connection within the clinical setting.^18^ Due to the relationship between obesity and CKD, bariatric surgery has been found to reduce the incidence and progression of obesity-related complications.^53,54^

In addition to bariatric surgery, pharmacological interventions, such as Orlistat, Liraglutide, Tirzepatide and semaglutides, have demonstrated weight loss efficiency and have been approved by Health Canada for weight loss.^55^ However, there is limited literature on the accessibility and utilization of these medications among Indigenous populations in Canada. However, it is known that glucagon-like peptide-1 receptor agonists (GLP1RA) offer kidney protection by slowing down and preventing CKD.^56^ Nevertheless, It is hypothesized that barriers such as limited healthcare access, medication cost, and lack of culturally competent care may also reduce the availability and uptake of these treatments among Indigenous communities. This barrier also influences kidney health with the recent advances in evidence supporting the use of GLP1RA to decrease the risk of CKD progression.^56^

### Gender Differences in Obesity

Gender differences in obesity prevalence and health behaviors are notable among Indigenous peoples. Women in some Indigenous communities are disproportionately affected by obesity, particularly abdominal obesity, which poses higher risks for cardiovascular diseases and metabolic disorders For example, Inuit women exhibit higher rates of abdominal obesity than their male counterparts, with central adiposity being strongly linked to metabolic risks such as hypertension and elevated triglyceride levels.^57^

Among Indigenous women, obesity is strongly associated with higher rates of gestational diabetes mellitus (GDM), which increases their own risk of developing type 2 diabetes (T2D) and also raises the risk of obesity and T2D in their children.^58,59^

Furthermore, socio-economic factors seem to impact obesity differently across genders. Indigenous women, particularly those with lower socio-economic status, face greater challenges in accessing healthy food and engaging in physical activities, contributing to higher obesity rates.^60^ In contrast, Indigenous men with higher socio-economic status are more likely to be obese, suggesting a complex interplay between income, gender, and health behaviors.^60^ These gender differences highlight the need for gender-sensitive interventions that address the specific barriers faced by Indigenous women and men in combating obesity, considering their distinct socio-economic realities and health risks.

Obesity is a key modifiable driver of CKD progression among Indigenous Peoples in Canada, acting both directly through mechanisms such as hyperfiltration and inflammation, and indirectly by accelerating the development of diabetes and hypertension resulting in a disproportionately higher burden of advanced CKD and end-stage kidney disease in this population.^61^

### Limited Access to Healthcare and Systemic Racism

Indigenous populations in Canada face significant barriers to healthcare access, particularly in rural and remote communities. For many Indigenous people, accessing healthcare often involves long travel distances, high costs, and inadequate transportation options.^62^ Additionally, healthcare services in these areas are frequently underfunded and understaffed, reducing the availability of timely and effective care.^63^ A critical gap exists in culturally competent care, with too few healthcare providers adequately trained to address the unique needs of Indigenous individuals.^63^ These structural inequalities in the healthcare system limit the effectiveness of obesity management and prevention services, contributing to the rising rates of obesity in Indigenous communities.^64^

Systemic racism and discrimination further exacerbate these healthcare barriers, playing a crucial role in the health disparities experienced by Indigenous peoples. Many Indigenous individuals report experiencing exclusion, derogatory treatment, and a lack of trust when interacting with healthcare providers.^64^ These encounters with racism discourage engagement with the healthcare system, leading to lower participation in obesity prevention programs and higher dropout rates.^64^ The historical trauma from colonization and the legacy of residential schools continues to shape Indigenous individuals’ relationships with healthcare, fostering reluctance to seek medical treatment, including care for obesity.^65^ Systemic racism also affects resource allocation, with Indigenous communities often receiving fewer resources and less culturally appropriate care, further perpetuating health inequalities and the prevalence of obesity.^66^

Current barriers to optimal kidney health in Indigenous Canadians include lack of primary care or specialist care and public health services; limited screening and patient tracking; and lack of culturally aware and safe care providers. Indeed, under this model, obesity is considered a second-hit factor in developing CKD.^67^ This applies in the later disease as well where Indigenous people on kidney replacement therapy (KRT) are less likely to receive a transplant despite the risk of CKD G5 being triple compared to the general population.^67^

### Education, Health Literacy and Policy Intervention

Many Indigenous communities, particularly in remote and rural areas, face barriers to accessing accurate and culturally relevant health information. Family members such as parents, grandparents, and elders are often the primary sources of health knowledge.^68^ While traditional health knowledge provides valuable insights, it may not fully address the multifactorial nature of modern medical challenges, such as the increasing prevalence of obesity. For instance, children in First Nations communities often associate traditional activities with health but lack a comprehensive understanding of obesity and its long-term effects.^68^ Although community-based health education programs have been implemented, the absence of sustained engagement with culturally tailored education reduces their overall effectiveness.

Despite efforts to incorporate Indigenous knowledge and traditional foods into education programs, these initiatives have sometimes failed to demonstrate significant improvements in health outcomes such as reductions in BMI or obesity rates.^69^ Programs that successfully increased health knowledge and shaped healthier dietary preferences have struggled to generate measurable decreases in obesity. This is partly due to misalignment with pressing local health concerns and the perception that these programs are externally imposed, lacking sufficient community involvement to drive lasting change.^69^

Low health literacy affects almost 25% of all CKD patients, with this being more prevalent in certain populations. This is associated with adverse clinical events, increased healthcare use, and mortality.^67^

Similarly, government policies and interventions targeting obesity in Indigenous peoples have often fallen short of achieving sustainable outcomes. While initiatives aimed at improving nutrition, physical activity, and food security have shown some success, they are frequently underfunded, short-term, and fail to address the root socio-economic causes of obesity.^66^ Many Indigenous communities remain underserved due to a lack of consistent and culturally appropriate policies. For instance, policies that address food affordability have had limited long-term success, particularly in remote areas, due to inconsistent funding and support.^70^ Store-based interventions in remote communities have resulted in healthier food purchases but sustaining these changes over time and translating successes to urban settings have proven challenging. The lack of a comprehensive, community-driven policy development approach has led to fragmented efforts that fail to tackle the complex, multifaceted causes of obesity in Indigenous populations.

Figure 2 presents a conceptual framework illustrating the interconnected determinants of obesity among Indigenous Peoples in Canada and their relationship to CKD risk and transplantation access.

**Figure 2.**
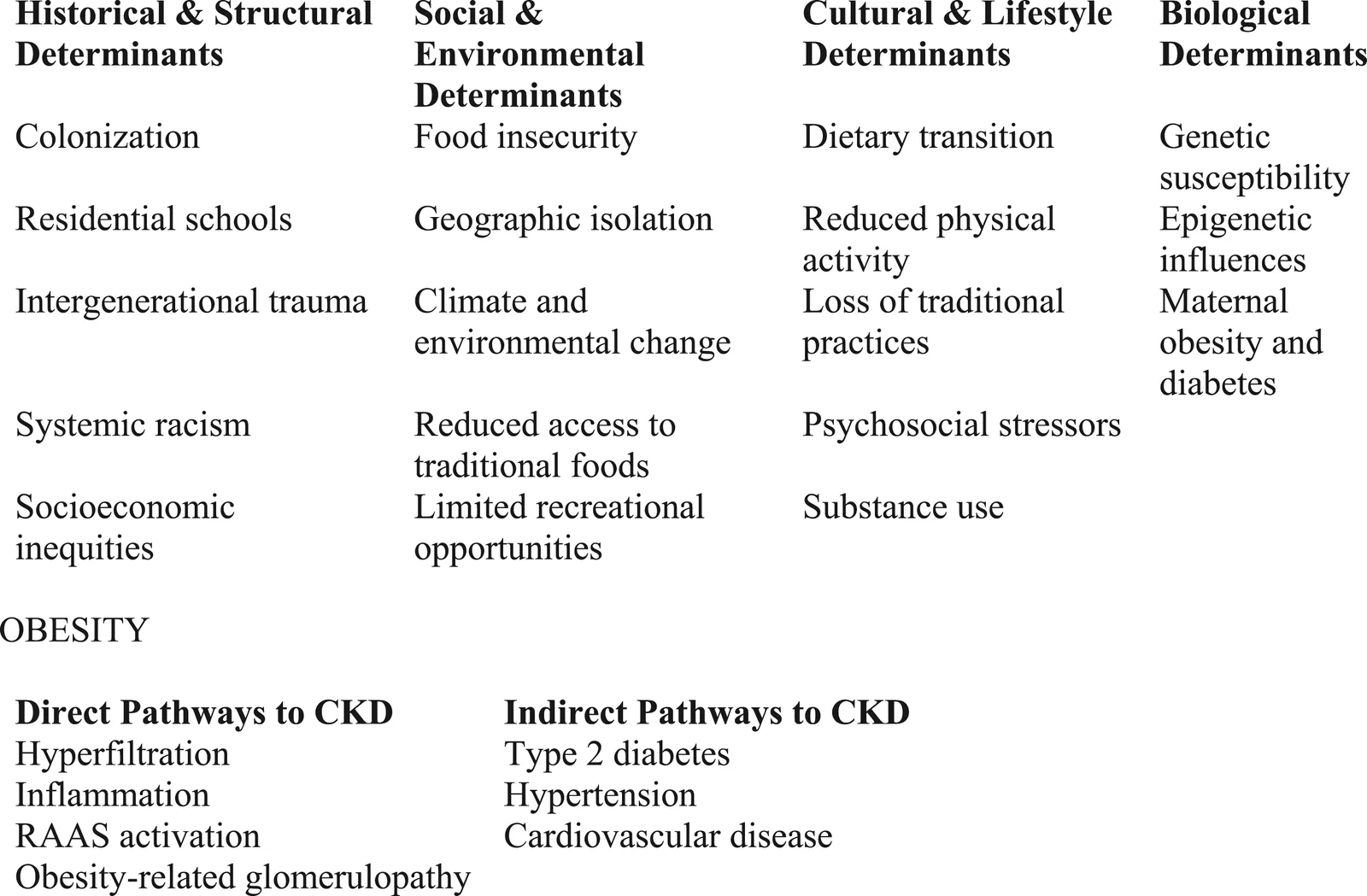
Conceptual framework of determinants of obesity and their relationship to chronic kidney disease among indigenous peoples in Canada obesity

## Chronic Kidney Disease

### Consequences

• Increased CKD progression
• Increased risk of kidney failure
• Reduced access to kidney transplantation
• Persistent health inequities

### Recommendations

It is recommended that obesity prevention and management among Indigenous Peoples in Canada prioritize the restoration of traditional food systems through support for hunting, fishing, and gathering, easing harvesting restrictions, and protecting wildlife habitats. It is further recommended that food security, particularly in remote and northern regions, be improved through targeted subsidies, local food production such as community gardens or greenhouses, and Indigenous-led food cooperatives. Culturally relevant physical activity should be promoted by integrating traditional land-based activities with investment in year-round, climate-adapted recreational facilities. Addressing socioeconomic inequities through targeted employment programs, income supports, and housing stability initiatives is also advised.

It is recommended that programs integrate trauma-informed mental health services and culturally grounded counselling to address coping mechanisms such as emotional eating, inactivity, and substance use. Healthcare access should be improved by increasing Indigenous representation in health professions, mandating cultural safety training, and expanding local care clinics with telehealth support. Bariatric surgery and pharmacological treatments should be made more accessible through financial coverage, streamlined processes, and culturally adapted pre- and post-treatment support. Interventions should also be gender-responsive to address the distinct needs and barriers faced by both women and men.

It is further recommended that obesity assessment tools be refined by adjusting BMI cutoffs and incorporating measures such as waist circumference and metabolic markers to reflect the unique health profiles of Indigenous populations. Finally, sustainable improvements require community-led initiatives that integrate traditional knowledge with evidence-based guidance, secure long-term funding, and align interventions with local priorities to achieve lasting reductions in obesity prevalence.

Future research should prioritize studies examining obesity as a primary, independent predictor of CKD in Indigenous Canadians beyond the diabetes-mediated pathway using multiple anthropometric measures rather than BMI alone. Longitudinal studies linking low birth weight to adult CKD risk and embedding CKD as an outcome in studies examining substance use, smoking, and sedentary behaviour, would address critical gaps. The toxic impact of heavy metals and persistent organic pollutants in traditional food systems on kidney outcomes also warrants dedicated investigation.

National expansion of Canada’s Kidney Check program with standardized obesity and waist circumference screening incorporated as core components is a cost-effective policy intervention. Additionally, subsidizing access to bariatric surgery and GLP-1 receptor agonists for Indigenous Canadians with obesity and CKD could directly improve kidney transplantation eligibility, addressing the inequity where Indigenous Canadians are less likely to receive a transplant.

## Limitations

The body of research examining obesity among Indigenous Peoples of Canada provides valuable insights, but there are several limitations that need to be addressed. Many studies rely on self-reported data for dietary intake, physical activity, and health outcomes, which introduces the risk of reporting bias and inaccuracies. Additionally, the reliance on cross-sectional designs limits the ability to establish causal relationships between contributing factors and obesity. The lack of longitudinal data further complicates the understanding of how these factors evolve over time and their long-term impact on health.

Moreover, much of the existing research is geographically specific, focusing on certain regions or communities, which limits the generalizability of the findings to other Indigenous populations across Canada. The scarcity of data on urban Indigenous populations, particularly those living off-reserve, highlights a critical gap in the literature.

Future research should prioritize longitudinal studies that examine the long-term effects of dietary, environmental, and socioeconomic changes on obesity in Indigenous populations. More research is needed to explore the impact of environmental contaminants on food systems and health, particularly in remote communities. Additionally, studies should consider more culturally appropriate methods of measuring health outcomes, as Western-centric tools may not fully capture Indigenous concepts of health and well-being.

## Conclusion

In conclusion, obesity among Indigenous Peoples of Canada is a significant and multifactorial contributor to the high burden of CKD in these communities. Driven by complex, interconnected factors such as dietary shifts, socioeconomic barriers, and systemic healthcare inequities, this challenge is further compounded by historical trauma, cultural disruption, and limited access to culturally appropriate care. Through its direct effects on kidney structure and function, as well as its strong association with diabetes and hypertension, obesity accelerates CKD onset and progression, limits treatment options, and reduces eligibility for kidney transplantation. Efforts to address obesity should be culturally grounded, community-driven, and sustained over time, focusing on improving access to healthy foods, promoting culturally relevant physical activity, reducing socioeconomic barriers, and ensuring the availability of effective obesity treatments. By prioritizing obesity reduction as a key strategy, we can help prevent CKD, improve eligibility for transplantation, and advance health equity for Indigenous Peoples in Canada.

## Supplemental Material

Supplemental material - Factors Contributing to Obesity Among Indigenous Peoples of Canada: A Narrative Review Supplemental material for Factors Contributing to Obesity Among Indigenous Peoples of Canada: A Narrative Review by Danial Aminaei, Minhal Ahmed, Asal Ozlati, Kristin Kyomi Clemens, Arsh Jain, Sonja Reichert, Stewart Harris, Jaclyn Ernst, and Louise Moist in Canadian Journal of Kidney Health and Disease.

Supplemental material - Factors Contributing to Obesity Among Indigenous Peoples of Canada: A Narrative Review Supplemental material for Factors Contributing to Obesity Among Indigenous Peoples of Canada: A Narrative Review by Danial Aminaei, Minhal Ahmed, Asal Ozlati, Kristin Kyomi Clemens, Arsh Jain, Sonja Reichert, Stewart Harris, Jaclyn Ernst, and Louise Moist in Canadian Journal of Kidney Health and Disease.

## References

[1] World Health Organization . Obesity and overweight [Internet]. Geneva: WHO. [cited 2025 Jun 2]. Available from. https://www.who.int/news-room/fact-sheets/detail/obesity-and-overweight

[2] Obesity Canada . Adult clinical practice guideline [Internet]. Edmonton, AB: Obesity Canada. [cited 2025 Jun 2]. Available from. https://obesitycanada.ca/healthcare-professionals/adult-clinical-practice-guideline/

[3] Anis AH Zhang W Bansback N , et al. Obesity and overweight in Canada: an updated cost of illness study. doi:10.1111/j.1467-789X.2009.00579.x, accessed 16 April 2026. 19413707

[4] Wharton S Lau DCW Vallis M , et al. Obesity in adults: a clinical practice guideline. CMAJ Can Med Assoc J. 2020;192:E875-E891. 10.1503/cmaj.191707 PMC7828878 32753461

[5] Wolin KY Carson K Colditz GA . Obesity and cancer. The Oncologist. 2010;15:556-565. 10.1634/theoncologist.2009-0285 PMC3227989 20507889

[6] Stasi A Cosola C Caggiano G , et al. Obesity-Related Chronic Kidney Disease: Principal Mechanisms and New Approaches in Nutritional Management. Front Nutr. 2022;9:925619. 10.3389/fnut.2022.925619 PMC9263700 35811945

[7] Kovesdy CP Furth SL Zoccali C . Obesity and kidney disease: hidden consequences of the epidemic. J Nephrol. 2017;30:1-10. 10.1007/s40620-017-0377-y 28214961

[8] Glicklich D Mustafa MR . Obesity in Kidney Transplantation: Impact on Transplant Candidates, Recipients, and Donors. Cardiol Rev. 2019;27:63-72. 10.1097/CRD.0000000000000216 29870421

[9] MacLaughlin HL Campbell KL . Obesity as a barrier to kidney transplantation: Time to eliminate the body weight bias? Semin Dial. 2019;32:219-222. 10.1111/sdi.12783 30941820

[10] Canada G of CC-IR and NA . Indigenous peoples and communities. 2009. https://www.rcaanc-cirnac.gc.ca/eng/1100100013785/1529102490303, accessed 16 April 2026.

[11] Batal M Decelles S . A Scoping Review of Obesity among Indigenous Peoples in Canada. J Obes. 2019;2019:9741090. 10.1155/2019/9741090 PMC6589240 31281674

[12] Komenda P Lavallee B Ferguson TW , et al. The Prevalence of CKD in Rural Canadian Indigenous Peoples: Results From the First Nations Community Based Screening to Improve Kidney Health and Prevent Dialysis (FINISHED) Screen, Triage, and Treat Program. Am J Kidney Dis Off J Natl Kidney Found. 2016;68:582-590. 10.1053/j.ajkd.2016.04.014 27257016

[13] Harasemiw O Milks S Oakley L , et al. Remote Dwelling Location Is a Risk Factor for CKD Among Indigenous Canadians. Kidney Int Rep. 2018;3:825-832. 10.1016/j.ekir.2018.02.002 PMC6035135 29989009

[14] Zacharias JM Young TK Riediger ND Roulette J Bruce SG . Prevalence, risk factors and awareness of albuminuria on a Canadian First Nation: A community-based screening study. BMC Public Health. 2012;12:290. 10.1186/1471-2458-12-290 PMC3403871 22521250

[15] Canadian Institute for Health Information . End-Stage Renal Disease Among Aboriginal Peoples in Canada: Treatment and Outcomes. Ottawa, ON: CIHI; 2013. Available from. https://publications.gc.ca/collections/collection_2013/icis-cihi/H117-5-17-2013-eng.pdf

[16] Truth and Reconciliation Commission of Canada . Truth and Reconciliation Commission of Canada: Calls to Action. Winnipeg, MB: TRC. 2015. Available from. https://trc.ca/assets/pdf/Calls_to_Action_English2.pdf

[17] Covidence: Better systematic review management [Internet]. Melbourne: Covidence; [cited 2025 Jun 2]. Available from. https://www.covidence.org/

[18] Akbar L Zuk AM Martin ID Liberda EN Tsuji LJS . Potential obesogenic effect of a complex contaminant mixture on Cree First Nations adults of Northern Québec, Canada. Environ Res. 2021;192:110478. 10.1016/j.envres.2020.110478 33212128

[19] Bruner BG Chad KE . Dietary practices and influences on diet intake among women in a Woodland Cree community. J Hum Nutr Diet Off J Br Diet Assoc. 2014;27(Suppl 2):220-229. 10.1111/jhn.12121 23662654

[20] Akande VO Fawehinmi TO Ruiter RAC Kremers SPJ . Healthy Dietary Choices and Physical Activity Participation in the Canadian Arctic: Understanding Nunavut Inuit Perspectives on the Barriers and Enablers. Int J Environ Res Public Health. 2021;18:940. 10.3390/ijerph18030940 PMC7908338 33499008

[21] Galloway T Blackett H Chatwood S , et al. Obesity studies in the circumpolar Inuit: a scoping review. Int J Circumpolar Health. 2012;71:18698. 10.3402/ijch.v71i0.18698 PMC4981754 22765938

[22] Bhawra J Cooke MJ Hanning R Wilk P Gonneville SLH . Community perspectives on food insecurity and obesity: Focus groups with caregivers of metis and Off-reserve first nations children. Int J Equity Health. 2015;14:96. 10.1186/s12939-015-0232-5 PMC4609156 26475264

[23] Domingo A Spiegel J Guhn M , et al. Predictors of household food insecurity and relationship with obesity in First Nations communities in British Columbia, Manitoba, Alberta and Ontario. Public Health Nutr. 2021;24:1021-1033. 10.1017/S1368980019004889 PMC8025097 32366338

[24] Cooke MJ Wilk P Paul KW Gonneville SLH . Predictors of Obesity Among Métis Children: Socio-economic, Behavioural and Cultural Factors. Can J Public Health. 2013;104:e298-e303. 10.17269/cjph.104.3765 PMC6973954 24044469

[25] Bhawra J Cooke MJ Guo Y Wilk P . The association of household food security, household characteristics and school environment with obesity status among off-reserve First Nations and Métis children and youth in Canada: results from the 2012 Aboriginal Peoples Survey. Health Promot Chronic Dis Prev Can Res Policy Pract. 2017;37:77-86. 10.24095/hpcdp.37.3.03 PMC5602162 28273035

[26] Johnson-Down L Egeland GM . Adequate Nutrient Intakes Are Associated with Traditional Food Consumption in Nunavut Inuit Children Aged 3–5 Years1,2. J Nutr. 2010;140:1311-1316. 10.3945/jn.109.117887 20444949

[27] Kerpan ST Humbert ML Henry CJ . Determinants of diet for urban aboriginal youth: implications for health promotion. Health Promot Pract. 2015;16:392-400. 10.1177/1524839914557862 25384578

[28] Huria T Pitama SG Beckert L , et al. Reported sources of health inequities in Indigenous Peoples with chronic kidney disease: a systematic review of quantitative studies. BMC Public Health. 2021;21:1447. 10.1186/s12889-021-11180-2 PMC8299576 34301234

[29] Harasemiw O Komenda P Tangri N . Addressing Inequities in Kidney Care for Indigenous People in Canada. J Am Soc Nephrol. 2022;33:1474-1476. 10.1681/ASN.2022020215 PMC9342645 35667707

[30] Ahmed F Zuk AM Tsuji LJS . The Impact of Land-Based Physical Activity Interventions on Self-Reported Health and Well-Being of Indigenous Adults: A Systematic Review. Int J Environ Res Public Health. 2021;18:7099. 10.3390/ijerph18137099 PMC8296996 34281031

[31] Togo F Watanabe E Park H Shephard RJ Aoyagi Y . Meteorology and the physical activity of the elderly: the Nakanojo Study. Int J Biometeorol. 2005;50:83-89. 10.1007/s00484-005-0277-z 16044348

[32] Foulds HJA Bredin SSD Warburton DER . An evaluation of the physical activity and health status of British Columbian Aboriginal populations. Appl Physiol Nutr Metab Physiol Appl Nutr Metab. 2012;37:127-137. 10.1139/h11-138 22269025

[33] Dyck RF Naqshbandi Hayward M Harris SB CIRCLE Study Group . Prevalence, determinants and co-morbidities of chronic kidney disease among First Nations adults with diabetes: results from the CIRCLE study. BMC Nephrol. 2012;13:57. 10.1186/1471-2369-13-57 PMC3438064 22776036

[34] Gates M Hanning RM Martin ID Gates A Tsuji LJS . Body Mass Index of First Nations youth in Ontario, Canada: influence of sleep and screen time. Rural Remote Health. 2013;13:2498. 24033103

[35] Fehderau DD Holt NL Ball GD , et al. Feasibility study of asset mapping with children: identifying how the community environment shapes activity and food choices in Alexander First Nation. Rural Remote Health. 2013;13:2289. doi:10.22605/RRH2289. Epub ahead of print 27 March 23534835

[36] Young TK Kaufert JM McKenzie JK Hawkins A O'Neil J . Excessive burden of end-state renal disease among Canadian Indians: a national survey. Am J Public Health. 1989;79:756-758. 10.2105/ajph.79.6.756 PMC1349637 2786348

[37] Gittelsohn J Harris SB Thorne-Lyman AL Hanley AJ Barnie A Zinman B . Body image concepts differ by age and sex in an Ojibway-Cree community in Canada. J Nutr. 1996;126:2990-3000. 10.1093/jn/126.12.2990 9001366

[38] Fee M . Racializing narratives: obesity, diabetes and the ‘Aboriginal’ thrifty genotype. Soc Sci Med. 2006;62:2988-2997. 10.1016/j.socscimed.2005.11.062 16426714

[39] Kue Young T Chateau D Zhang M . Factor analysis of ethnic variation in the multiple metabolic (insulin resistance) syndrome in three Canadian populations. Am J Hum Biol Off J Hum Biol Counc. 2002;14:649-658. 10.1002/ajhb.10083 12203819

[40] Schafte K Bruna S . The influence of intergenerational trauma on epigenetics and obesity in Indigenous populations - a scoping review. Epigenetics. 2023;18:2260218. 10.1080/15592294.2023.2260218 PMC10538456 37752750

[41] Haman F Batal M Imbeault P Blais JM Robidoux MA . Obesity and Type 2 Diabetes in Northern Canada’s Remote First Nations Communities: The Dietary Dilemma. Int J Obes. 2010;34(Suppl 2):S24-S31. 10.1038/ijo.2010.236 21151143

[42] Sharp D . Environmental toxins, a potential risk factor for diabetes among Canadian Aboriginals. Int J Circumpolar Health. 2009;68:316-326. 10.3402/ijch.v68i4.17372 19917184

[43] Sheikh N Egeland GM Johnson-Down L Kuhnlein HV . Changing dietary patterns and body mass index over time in Canadian Inuit communities. Int J Circumpolar Health. 2011;70:511-519. 10.3402/ijch.v70i5.17863 22152598

[44] Atlantic Centre of Excellence for Women's Health . Aboriginal Women and Obesity in Canada: A Review of the Literature. Halifax, NS: ACEWH; 2009. Available from. https://cdn.dal.ca/content/dam/dalhousie/pdf/diff/ace-women-health/live/ACEWH_aboriginal_women_obesity_canada_lit_review_July_09.pdf

[45] Hegele RA Cao H Harris SB Zinman B Hanley AJ Anderson CM . Gender, obesity, hepatic nuclear factor-1alpha G319S and the age-of-onset of type 2 diabetes in Canadian Oji-Cree. Int J Obes Relat Metab Disord J Int Assoc Study Obes. 2000;24:1062-1064. 10.1038/sj.ijo.0801258 10951547

[46] Château-Degat M-L Dewailly E Charbonneau G Laouan-Sidi EA Tremblay A Egeland GM . Obesity risks: towards an emerging Inuit pattern. Int J Circumpolar Health. 2011;70:166-177. 10.3402/ijch.v70i2.17802 21524362

[47] Charbonneau-Roberts G Saudny-Unterberger H Kuhnlein HV Egeland GM . Body mass index may overestimate the prevalence of overweight and obesity among the Inuit. Int J Circumpolar Health. 2005;64:163-169. 10.3402/ijch.v64i2.17969 15945286

[48] Chiolero A Faeh D Paccaud F Cornuz J . Consequences of smoking for body weight, body fat distribution, and insulin resistance. Am J Clin Nutr. 2008;87:801-809. 10.1093/ajcn/87.4.801 18400700

[49] Kwok A Dordevic AL Paton G Page MJ Truby H . Effect of alcohol consumption on food energy intake: a systematic review and meta-analysis. Br J Nutr. 2019;121:481-495. 10.1017/S0007114518003677 30630543

[50] Fazzino TL Fleming K Sher KJ Sullivan DK Befort C . Heavy Drinking in Young Adulthood Increases Risk of Transitioning to Obesity. Am J Prev Med. 2017;53:169-175. 10.1016/j.amepre.2017.02.007 PMC5522652 28365088

[51] Whyte M Daeninck F Linton J , et al. Experiences and Outcomes of Indigenous Patients Undergoing Bariatric Surgery: a Mixed-Method Scoping Review. Obes Surg. 2024;34:1343-1357. 10.1007/s11695-024-07089-5 38400946

[52] Browne AJ Varcoe C Lavoie J , et al. Enhancing health care equity with Indigenous populations: evidence-based strategies from an ethnographic study. BMC Health Serv Res. 2016;16:544. 10.1186/s12913-016-1707-9 PMC5050637 27716261

[53] Lovrics O Doumouras AG Gmora S Anvari M Hong D . Metabolic outcomes after bariatric surgery for Indigenous patients in Ontario. Surg Obes Relat Dis. 2019;15:1340-1347. 10.1016/j.soard.2019.05.035 31300284

[54] Hossain I O’Brien E Dogar I Dubinsky I Pace D Smith C . Outcomes and experiences of Indigenous patients in Newfoundland and Labrador’s bariatric surgery program: a pilot study. Can J Surg J Can Chir. 2025;68:E169-E174. 10.1503/cjs.000125 PMC12114114 40399034

[55] Health Canada . Prescription Drugs Approved for Weight Management. Ottawa, ON: Government of Canada; 2021. [cited 2025 Jun 2]. Available from. https://www.canada.ca/en/health-canada/services/

[56] Perkovic V Tuttle KR Rossing P , et al. Effects of Semaglutide on Chronic Kidney Disease in Patients with Type 2 Diabetes. N Engl J Med. 2024;391:109-121. 10.1056/NEJMoa2403347 38785209

[57] Young TK . Obesity, central fat patterning, and their metabolic correlates among the inuit of the central Canadian Arctic. Hum Biol. 1996;68:245-263. 8838915

[58] Dyck RF Karunanayake C Pahwa P Stang M Osgood ND . Epidemiology of Diabetes in Pregnancy Among First Nations and Non-First Nations Women in Saskatchewan, 1980‒2013. Part 1: Populations, Methodology and Frequencies (1980‒2009); Results From the DIP: ORRIIGENSS Project. Can J Diabetes. 2020;44:597-604. 10.1016/j.jcjd.2019.10.005 31987766

[59] Willows ND Hanley AJG Delormier T . A socioecological framework to understand weight-related issues in Aboriginal children in Canada. Appl Physiol Nutr Metab Physiol Appl Nutr Metab. 2012;37:1-13. 10.1139/h11-128 22269027

[60] Canadian Research Data Centre Network . Obesity among off-reserve First Nations, Métis and Inuit peoples in Canada's provinces: associated factors and secular trends. [Internet]. Ottawa, ON: CRDCN; [cited 2025 Jun 2]. Available from. https://crdcn.ca/publication/obesity-among-off-reserve-first-nations-metis-and-inuit-peoples-in-canadas-provinces-associated-factors-and-secular-trends/

[61] Kelly L Matsumoto C Schreiber Y , et al. Prevalence of chronic kidney disease and cardiovascular comorbidities in adults in First Nations communities in northwest Ontario: a retrospective observational study. CMAJ Open. 2019;7:E568-E572. 10.9778/cmajo.20190040 PMC6768774 31501170

[62] Davy C Harfield S McArthur A Munn Z Brown A . Access to primary health care services for Indigenous peoples: A framework synthesis. Int J Equity Health. 2016;15:163. 10.1186/s12939-016-0450-5 PMC5045584 27716235

[63] Nguyen NH Subhan FB Williams K Chan CB . Barriers and Mitigating Strategies to Healthcare Access in Indigenous Communities of Canada: A Narrative Review. Healthcare. 2020;8:112. 10.3390/healthcare8020112 PMC7349010 32357396

[64] Kumanyika SK . Unraveling common threads in obesity risk among racial/ethnic minority and migrant populations. Public Health. 2019;172:125-134. 10.1016/j.puhe.2019.04.010 PMC6698150 31227270

[65] Mason CW McHugh T-LF Strachan L Boule K . Urban indigenous youth perspectives on access to physical activity programmes in Canada. Qual Res Sport Exerc Health. 2019;11:543-558.

[66] Office of the Provincial Health Officer . Indigenous Health and Well-being: Final Update. Victoria, BC: Government of British Columbia; 2018. Available from. https://www2.gov.bc.ca/assets/gov/health/about-bc-s-health-care-system/office-of-the-provincial-health-officer/reports-publications/special-reports/indigenous-health-well-being-2018.pdf

[67] Tungsanga S Okpechi IG Chaturvedi S , et al. Inequities in the Care and Outcomes of Indigenous People Living With Kidney Failure. Adv Kidney Dis Health. 2025;32:279-289. 10.1053/j.akdh.2024.10.008 40818893

[68] Pigford A-AE Willows ND Holt NL Newton AS Ball GDC . Using first nations children’s perceptions of food and activity to inform an obesity prevention strategy. Qual Health Res. 2012;22:986-996. 10.1177/1049732312443737 22645224

[69] Norman M Petherick L Garcia E Giesbrecht G Duhamel T . Governing indigenous recreation at a distance: a critical analysis of an after school active health intervention. Sport Educ Soc. 2016;23:1-14.

[70] Browne J Lock M Walker T Egan M Backholer K . Effects of food policy actions on Indigenous Peoples’ nutrition-related outcomes: a systematic review. BMJ Glob Health. 2020;5:e002442. 10.1136/bmjgh-2020-002442 PMC7437701 32816952

