# Supplementary material for: Factors Contributing to Obesity Among Indigenous Peoples of Canada: A Narrative Review
Source: Can J Kidney Health Dis. 2026 Sep 30;13:20543581261472119. doi: 10.1177/20543581261472119 (PMC13770002; doi:10.1177/20543581261472119)
Supplement: Supplementary file 1 [file sj-pdf-1-cjk-10.1177_20543581261472119.pdf]

CJKHD-25-0090.R3

Title: Factors Contributing to Obesity Among Indigenous Peoples of Canada: A Narrative Review

Résumé

Motif de la revue
L’obésité est un problème de santé publique majeur et en progression au Canada, et constitue un important facteur de risque pour l’insuffisance rénale chronique (IRC). Au Canada, les peuples autochtones sont touchés de façon disproportionnée par l’obésité et l’IRC en raison de facteurs sociaux, culturels, environnementaux et biologiques complexes. Cette revue résume la documentation existante sur les facteurs contribuant à l’obésité dans cette population et discute des répercussions sur la santé rénale.

Méthodologie
Une revue narrative a été réalisée en utilisant des termes de recherche prédéfinis associés à l’obésité, aux peuples autochtones du Canada et à l’IRC. Après une sélection des articles admissibles dans Covidence (n=131), 44 études ont été retenues en raison de leur pertinence et de leur contribution aux thèmes émergents.

Principales observations
Chez les peuples autochtones du Canada, l’obésité résulte d’un ensemble de facteurs interreliés, notamment la transition alimentaire, l’insécurité alimentaire, les inégalités socioéconomiques, le faible niveau d’activité physique, les changements environnementaux, les perturbations des repères culturels, les traumatismes historiques, le racisme systémique et l’accès limité à des soins de santé culturellement sécuritaires. Des facteurs biologiques et génétiques pourraient également y contribuer. Ces facteurs accroissent le risque d’IRC en entraînant directement des lésions rénales liées à l’obésité et, indirectement, le diabète et l’hypertension. Les obstacles à la prise en charge de l’obésité, notamment l’accès restreint à la chirurgie bariatrique, aux traitements pharmacologiques et à des soins culturellement adaptés, pourraient aggraver les disparités en santé rénale et en matière d’accès à la transplantation.

Limites
La documentation existante repose principalement sur des données transversales et autodéclarées. Les résultats sont limités par le manque d’études longitudinales, la sous-représentation des populations autochtones urbaines et la faible évaluation de mesures de santé et d’obésité culturellement pertinentes.

Conclusion
La prise en charge de l’obésité chez les peuples autochtones nécessite des interventions ancrées dans les réalités culturelles, dirigées par les communautés et déployées à l’échelle des systèmes, afin d’améliorer la sécurité alimentaire, l’accès aux soins et la prise en charge de l’obésité dans un contexte culturellement sécurisant. De telles approches pourraient contribuer à réduire le fardeau de l’IRC et favoriser l’équité en santé rénale.
